# Evaluating the Functionality of Conjunctiva Using a Rabbit Dry Eye Model

**DOI:** 10.1155/2016/3964642

**Published:** 2016-03-20

**Authors:** Yuan Ning, Dhruva Bhattacharya, Richard E. Jones, Fangkun Zhao, Rongji Chen, Jinsong Zhang, Mingwu Wang

**Affiliations:** ^1^Department of Ophthalmology, The Fourth Affiliated Hospital of China Medical University, Eye Hospital of China Medical University, Key Lens Research Laboratory of Liaoning Province, Shenyang 115001, China; ^2^Department of Ophthalmology and Vision Science, University of Arizona College of Medicine, Tucson, AZ 85711, USA

## Abstract

*Purpose.* To assess the conjunctival functionality in a rabbit dry eye (DE) model.* Methods.* Nictitating membrane, lacrimal and Harderian glands were surgically excised from male New Zealand white rabbits using minimally invasive surgery. Fluorescein/rose Bengal staining of ocular surface (OS) and Schirmer test were done before (BE) and after excision (AE). The expression of interleukin- (IL-) 1*β*, tumor necrosis factor- (TNF-) *α*, and MUC5AC proteins were estimated by immunoblotting from conjunctival impression cytology specimens. MUC5AC mRNA was quantified as well. The effect of epithelial sodium channel (ENaC) blockers on tear production and potential differences (PD) of OS were assessed under anesthesia in rabbits with and without surgery.* Results.* Increase in corneal and conjunctival staining was observed 1 month AE compared to BE. Schirmer tests failed to show decrease in tear production. Elevated IL-1*β*, and TNF-*α*, 1 month AE indicated inflammation. MUC5AC expression was elevated 1 month AE. ENaC blockers did not improve tear production in rabbit eyes AE but characteristic changes in PD were observed in rabbits with surgery.* Conclusions.* DE biomarkers are important tools for OS assessment and MUC5AC expression is elevated in rabbit DE. PD measurement revealed significant electrophysiological changes in rabbits with surgery.

## 1. Introduction

Tear film (TF) constantly protects the exposed surface of the eye, the cornea, and the conjunctiva from environmental stresses including desiccation, temperature change, physical injury, and infections [1]. By providing optimal concentrations of electrolytes, proteins, mucin, and lipids, the TF is critical in the maintenance of corneal transparency and good vision [1]. Dry eye disease (DED) is a multifactorial dysfunction of the TF, resulting in symptoms of discomfort, visual disturbance, and even loss of vision due to damage to the ocular surface [2]. DED is generally acknowledged to be, in large part, due to reduced secretion or increased evaporation of the tear fluid, resulting in subsequent increase in osmolarity and inflammation at the ocular surface [2]. Since DED represents a diverse group of conditions that manifest as inadequate ocular surface lubrication, restoration of a sufficient tear volume remains the mainstay of current dry eye (DE) treatment.

Although lacrimal gland (LG) is considered the main source of tears [3], increasing evidence suggests that under certain conditions conjunctival epithelium has the capacity to be the primary source of TF [1]. Removal of the main LG of squirrel monkeys does not lead to keratoconjunctivitis sicca (KCS) [4]. In humans, up to 86% of patients with epiphora who underwent palpebral dacryoadenectomy (PDA) did not develop DE, and in up to 50% of such patients the epiphora persisted [5, 6]. Although accessory LGs were believed to be mostly responsible in these cases, the conjunctiva certainly plays a role as a compensatory tissue. The human conjunctiva occupying 17 times more surface area than the cornea has the potential to be the primary modulator of tear volume and component [7].

We are interested in understanding the physiology of conjunctival epithelium so as to maximize its fluid secretion capacity as an alternative to DED treatment. A rabbit model with intact conjunctiva and equal DE phenotype bilaterally is ideal in such research. We created a DE model in rabbits by surgical excision of the nictitating membrane (NM), Harderian gland (HG), and main LG [8]. Surprisingly, the tear secretion was not significantly reduced by these operations. Although DE associated ocular surface phenotype and inflammatory biomarkers elevated in the immediate postoperative period, they gradually decreased over 4-month duration to near preoperative level without therapeutic intervention [8]. These findings suggest that the rabbit ocular surface can potentially compensate for the loss of these seemingly vital ocular surface structures, including the main LG. The results also indicate that, in acute DE condition (as created in our experiment), ocular surface injury and inflammation can be mostly reverted. To gain further insight into the exact mechanisms of conjunctiva mediated tear compensation, the present study further explored methods of conjunctival characterization in this mixed mechanism rabbit DE model.

## 2. Methods

### 2.1. Experimental Animals and Ethics Statement

Male New Zealand white rabbits (*N* = 8, 16 eyes, Harlan Sprague Dawley, Indianapolis, IN, USA) weighing 2.0–2.5 kg were used for this study. The rabbits were reared under standard laboratory conditions (22 ± 2°C, 40% ± 5% relative humidity, and a 12-hour light-dark cycle) with free access to food and water throughout the experiment. The study was conducted in compliance with the Tenets of the Declaration of Helsinki and ARVO statement for the use of animals in ophthalmic and visual research. The protocol was approved by the University of Arizona (Tucson, AZ, USA) Institutional Animal Care and Use Committee (protocol# 14-511). All surgeries were performed by skilled surgeons (YN and MW).

### 2.2. Operative Procedure

The surgical protocol for resection of main LG, HG, and NM was published previously [8] which was modified from established procedures [9, 10]. Identical procedure was performed on the left and right eye.

### 2.3. Evaluations

The rabbits were assessed before excision (BE) and after excision (AE). To minimize slit lamp finding artifact from other tests, the evaluations were carried out in two days in the following sequence of each eye. The first day begins with corneal fluorescein test, followed immediately by rose Bengal staining and CIC. On the second day, Schirmer tests, without (Schirmer I test, SIt) and with anesthesia (Schirmer II test, SIIt), were performed separately in the morning and afternoon.

### 2.4. Corneal Fluorescein and Rose Bengal Staining Tests

The eyes of all rabbits were examined under a slit lamp microscope (GR-54, Gilras LLC, Miami, FL) by the same ophthalmologist (YN) following protocol described previously [8].

### 2.5. Schirmer I and II Tests

Both SIt and SIIt were carried out in our study. The SIt was performed as per the protocol described previously [8]. For SIIt, one drop of 0.5% proparacaine hydrochloride (Bausch and Lomb, Tampa, FL, USA) was placed and the excess fluid was blotted away with soft paper tissue, prior to the insertion of the filter paper strips (Alcon Laboratories, Inc., Fort Worth, TX, USA) in the lower lateral one-third of conjunctival fornix and eyelids closed by gentle force for 5 mins. Both tests were performed three times and the average score was used for analysis.

### 2.6. Conjunctival Impression Cytology

Conjunctival impression cytology (CIC) was performed as per the protocol published [8]. The filter paper discs were peeled off and immediately placed in either 500 *μ*L Trizol solution (Invitrogen, CA, USA) for RNA isolation or 100 *μ*L of radio immunoprecipitation assay (RIPA) buffer (Teknova, CA, USA) for protein isolation.

### 2.7. RNA Isolation and cDNA Synthesis

Total RNA was isolated from the CIC specimens in Trizol solution according to manufacturer's instructions (Invitrogen, CA, USA). RNA concentrations were estimated by NanoDrop ND-1000 Spectrophotometer (NanoDrop Technologies, Wilmington DE, USA) in 1 *μ*L volume. Purity of the RNA was assessed by the ratio of absorbance at 260/280 nm. A ratio of 1.9 to 2 was considered to be good quality RNA specimen and used for further experiments. The first strand of cDNA was synthesized with QuantiTect® Reverse Transcription Kit (Qiagen, Valencia, CA, USA) using 500 ng total RNA according to the manufacturer's instructions.

### 2.8. Reverse Transcriptase-Quantitative Polymerase Chain Reaction (RT-qPCR)

The RT-qPCR reactions were set using SYBR® Green PCR Master Mix (Applied Biosystems, Foster City, CA, USA) according to manufacturer's instructions. The primer sequences for MUC5AC were as follows: MUC5AC-F: CCCCAACGTCAAGAACAACT and MUC5AC-R: TCAAACAGGCAGTTCGAGTG [11]. The RT-qPCR was performed on StepOnePlus*™* Real-Time PCR System (Applied Biosystems) with the following cycling conditions: 15 min 95°C, 40 cycles of 15 sec 95°C, and 30 sec 60°C. The fluorescence was recorded during elongation step in each cycle. A melting curve analysis was performed at the end of each PCR by gradually increasing the temperature from 60 to 95°C while recording the fluorescence. A single peak at the melting temperature of the PCR product confirmed primer specificity. To compare between different runs, a fixed fluorescence threshold for derivation of C_T_ value for all runs was used. Three technical replicates were performed to evaluate the relative quantification.

### 2.9. Relative Quantification of mRNA Level

Relative quantification of MUC5AC expression in rabbit CIC specimens was performed BE and 1 month AE. The fold change in MUC5AC expression was relative to the internal housekeeping gene, *β*-actin (endogenous control). Mean fold change in MUC5AC expression was calculated using 2^−ΔΔC_T_^ method, where ΔΔC_T_ = (C_T__Gene_ − C_T__Actin_)_After  Excision_ − (C_T__Gene_ − C_T__Actin_)_Before  Excision_. Difference between C_T_ for MUC5AC and *β*-actin mRNA in each specimen was used to calculate level of target mRNA relative to that of *β*-actin mRNA in the same specimen [8].

### 2.10. Immunoblotting

Total cell lysate proteins were isolated from CIC in radio immunoprecipitation assay (RIPA) buffer with 1x HALT protease and phosphatase inhibitor single use inhibitor cocktail (Thermoscientific, Rockford, IL, USA) by incubating on ice for 30 min. Protein concentration was determined by Pierce*™* BCA Protein Assay Kit (Thermo Fisher Scientific, NY). Specimens were mixed with Laemmli sample buffer (Bio-Rad laboratories, Inc. Hercules, CA, USA) containing *β*-mercaptoethanol and heated at 95°C for 10 min. Specimens were then immunoblotted and analyzed as per the protocol published previously [8]. The primary rabbit monoclonal antibodies to IL-1*β*, TNF-*α*, and MUC5AC (Abcam, Cambridge, MA, USA) were used at a dilution of 1: 200.

### 2.11. Effect of Epithelial Sodium Channel Blockers on Conjunctival Tear Secretion

To test the effects of epithelial sodium channel (ENaC) blockers on tear secretion, amiloride and benzamil were administered topically to the right eyes (*n* = 8) of the operated rabbits 2 months AE. A 0.1% of amiloride and benzamil [12] (both from Sigma-Aldrich, Inc. St. Louis, MO, USA) were prepared in sterile buffered saline solution (BSS) and tested in separate experiments. The right eyes were allocated to ENaC blockers and left eyes to BSS as control. A 50 *μ*L of ENaC blocker eye drops or BSS was instilled into the lower conjunctival sac by a micropipette at the beginning of the experiments. SIIt was performed before and at 5 min, 15 min, 30 min, 60, and 90 min after application of amiloride or benzamil.

### 2.12. Open-Circuit Potential Difference Measurements at the Rabbit Ocular Surface

Potential difference (PD) is generated by electrogenic Cl^−^ secretion and Na^+^ reabsorption across superficial cell apical membrane of the corneal and conjunctival epithelia [13]. PD measurement is a sensitive modality in detecting transepithelial electrolyte conductance at the ocular surface [14]. Therefore, to help delineate the underlying physiological change which contributes to the increased output of tears by the rabbit conjunctiva AE, open-circuit PD was measured with a method modified from a previously established protocol in mice [14]. Briefly, the rabbits were anesthetized with 100 mg/kg ketamine and 10 mg/kg xylazine (Sigma-Aldrich, MO, USA) and placed on a heating pad in a stereotaxic device with conjunctival and corneal tissues exposed and faced upwards. Two different solutions were perfused in series over the ocular surface at a rate of 10 mL/min using a pinch valve perfusion system (PS-8H; Bioscience Tools, San Diego, CA, USA) and peristaltic pump (13-876-1; Fisher Scientific, Pittsburgh, PA, USA) with 1/16′′ inner-diameter plastic tubing.

First, phosphate-buffered saline (1x PBS) was perfused for 5 min to establish a stable baseline, and then 100 *μ*M amiloride (Sigma-Aldrich) prepared in 1x PBS was perfused. A low powered wall vacuum attached to 1/16′′ ID tubing was placed next to the fluid bolus covering the ocular surface to keep the volume constant and avoid fluid runoff. The PDs were measured with a high-impedance digital voltmeter, IsoMilivolt Meter (World Precision Instruments, Sarasota, FL, USA) with two Ag/AgCl electrodes connected through a 1 M KCl agar bridge. One probe was placed in contact with the ocular fluid, and the other was placed subcutaneously in the rabbit's mid-back. The PDs were measured on operated rabbit eyes (*n* = 4) 5 months AE and compared with normal rabbit eyes (*n* = 4) as controls.

### 2.13. Data Analysis and Statistics

Data in figures are presented as mean Standard Error Method, the bars representing standard errors. Statistical significance between two groups (BE and AE) was evaluated using unpaired 2-tailed *t*-test. A probability of *P* equal to 0.05 was considered significant (where applicable, ^*∗*^ *P* < 0.05, ^*∗∗*^ *P* < 0.01, and ^*∗∗∗*^ *P* < 0.001). The Spearman correlation analysis was employed to determine the correlation between every pair of the tests performed, BE and AE.

## 3. Results

### 3.1. Modification of the Operative Procedure

**Figure 1:**
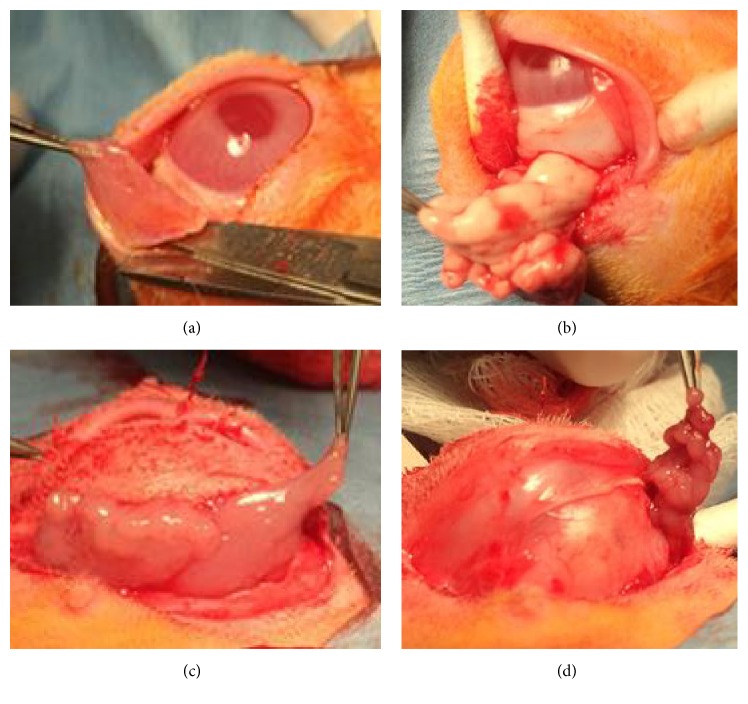
Major surgical steps involved in creating our rabbit dry eye model. (a) Nictitating membrane (NM) was removed at the base; (b) Harderian gland was separated and ablated through same wound as excision of NM (this was done to reduce hemorrhage); (c) removal of infraorbital and temporal lobes of the lacrimal gland; (d) removal of the deeply embedded intraorbital lobe of the lacrimal gland.

Chen et al. extracted the HG through an inferior orbital rim incision [9]. We found that this approach requires a long incision toward the medial canthus. In addition, massive hemorrhage tends to occur while excising the HG from between the medial rectus muscle and the anterior orbital wall. Gelatin sponge was used to achieve hemostasis during their surgeries. In our study, excision of the HG through the NM excision wound was much less invasive. Less hemorrhage and improved visibility of the surgical field ensured complete ablation of the HG (Figure 1). Li et al. extracted HG using similar method [10]. However, a 5 mm palpebral conjunctival incision was made in their study to extract lobes of the main LG. In our experience, such a small incision would not permit adequate access to all lobes of the LG, especially the intraorbital lobe, which is deeply embedded beneath the lateral orbital rim and separated by a dense membranous connective tissue from the superficial temporal lobe. No additional conjunctival incision was necessary in our procedure and hence the entire conjunctival surface is preserved. The skin incision only needed to cover the lateral two-thirds of the orbital rim in order to have a good exposure to adequately remove the infraorbital, temporal, and intraorbital lobes of the LG. A rabbit model with intact conjunctiva and equal DE phenotype bilaterally is ideal for our research to comparatively assess modalities that potentially stimulate conjunctival fluid secretions.

### 3.2. Ocular Surface Changes

**Figure 2:**
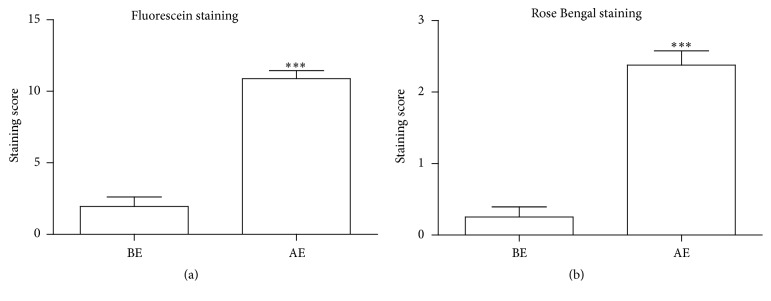
Comparison of fluorescein and rose Bengal staining of rabbit eyes before and 1 month after surgery. There were significant differences in fluorescein staining (a) and rose Bengal staining (b) (^*∗∗∗*^ *P* < 0.0001) before excision (BE) and after excision (AE). Data are presented as mean Standard Error Method (SEM).

As compared to BE, both fluorescein and rose Bengal staining increased on the cornea and conjunctiva (Figure 2) 1 month AE. Significantly higher staining scores (*P* < 0.0001 in both) demonstrated the presence of DE phenotype at the ocular surface. For all tests conducted, there were no significant differences found as a function of left versus right eye.

### 3.3. Schirmer's Tests

**Figure 3:**
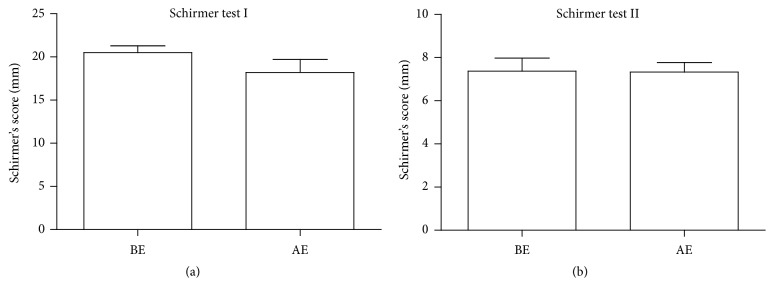
Comparison of Schirmer tests (I and II) before (BE) and 1 month after excision (AE). There were no significant differences in Schirmer scores BE and 1 month AE, either without anesthesia (Schirmer I) or with anesthesia (Schirmer II). Data are presented as mean Standard Error Method (SEM).

In our study, large variations were noted in both Schirmer tests among eyes tested either BE or AE. With both SIt (*P* = 0.104) and SIIt (*P* = 0.478), no significant reduction in tear production was seen 1 month AE (Figure 3). There was, however, significant difference between the SIt and SIIt (*P* < 0.0001) either BE or AE, with tear secretion being lower under topical anesthesia.

### 3.4. Upregulation of Dry Eye Biomarkers

**Figure 4:**
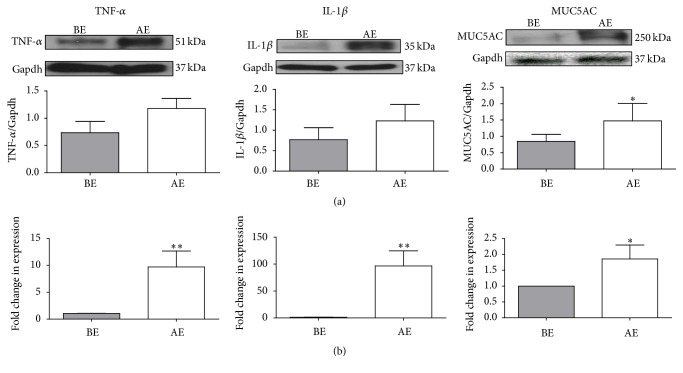
Quantification of mRNA and protein levels of inflammatory cytokines and MUC5AC in rabbit conjunctival impression cytology specimens BE and 1 month AE. For the proteins, the signal for the gene was normalized with the Gapdh signal from the same gene (a). For mRNA, the fold change in expression of genes is relative to endogenous control, *β*-actin (b). The data for mRNA of IL-1*β* and TNF-*α* (b) were referred from the previous publication [8]. The upregulation of mRNA correlated with the increase at protein level for the inflammatory cytokines and MUC5AC. Data are presented as mean Standard Error Method (SEM). For all graphs, bars show standard error (SE); statistical differences are shown (^*∗*^ *P* < 0.05, ^*∗∗*^ *P* < 0.01).

The protein levels of DED associated inflammatory cytokines (TNF-*α* and IL-1*β*) increased 1 month AE (Figure 4) which corroborated with the mRNA levels of the inflammatory cytokines as reported previously [8]. Increase of conjunctival epithelium encoded goblet cell-specific MUC5AC at mRNA and protein levels were observed 1 month AE (Figure 4).

### 3.5. Effect of Amiloride and Benzamil Treatment on Conjunctiva Secretion

**Figure 5:**
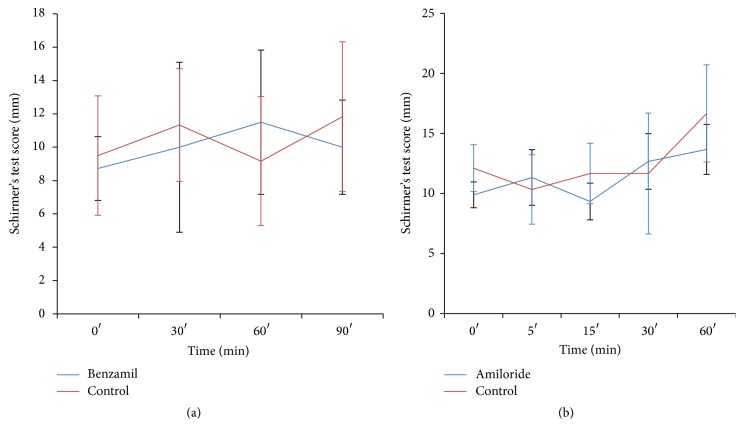
Effect of epithelial sodium channel blockers on rabbit DE model. The application of epithelial sodium channel blockers, benzamil (a) and amiloride (b), did not significantly increase the tear quantity in our rabbit DE model.

The two ENaC blockers did not increase tear secretion in our rabbit DE model as measured by SIIt (Figure 5).

### 3.6. Open-Circuit Potential Difference and Depolarization after Amiloride Treatment at the Ocular Surface of Rabbits

**Figure 6:**
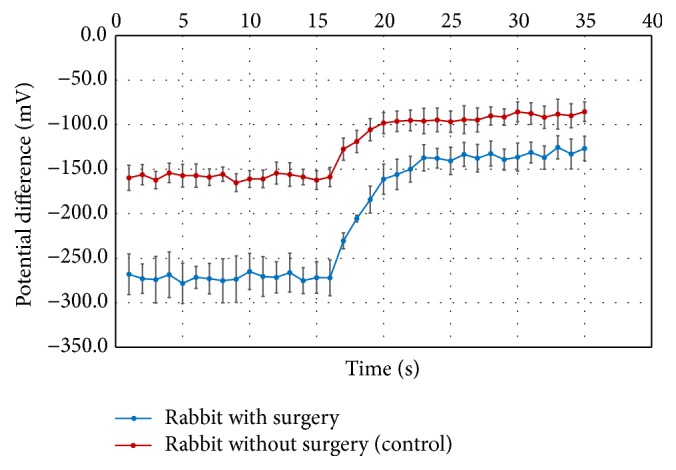
Potential difference recordings of the rabbit eyes after surgery compared to rabbit eyes without surgery. The potential differences were recorded for the rabbit eyes 5 months AE (*n* = 4) and control eyes (*n* = 4). The perfusion channel was switched from PBS to amiloride at 13 seconds, with 2-3 seconds required for the new solution to reach the ocular surface. Data are presented as mean Standard Error Method (SEM).

The PD measurements for the 10 seconds before the perfusion system was switched from PBS to amiloride channel were −272 ± 6 mV for rabbit eyes in the operated group (*n* = 4) and −159 ± 3 mV for the control group (*n* = 4). The difference in PDs was highly significant (*P* < 0.005). After the ocular surface was perfused with amiloride, the 10-second average PD reached −133 ± 4 mV in the operated eyes and −90 ± 4 mV in the control eyes. The magnitude of depolarization was statistically larger (*P* < 0.05) in the operated eyes than in the control eyes (Figure 6).

### 3.7. Statistical Correlations between Various Tests

Using Spearman correlation analysis, higher SIIt scores are closely associated with lower rose Bengal test scores (negatively correlated, correlation coefficient = −0.57, *P* = 0.02). Additionally, RT-qPCR of IL-1*β* and TNF-*α* were significantly correlated (correlation coefficient = 0.72, *P* = 0.02). The changes of inflammatory biomarkers did not correlate with that of the clinical tests (fluorescein staining, rose Bengal staining, and Schirmer tests).

## 4. Discussion

In our study, as expected, the rabbits showed increased fluorescein and rose Bengal staining of the ocular surface 1 month AE, characteristic of DE phenotype. Interestingly, no significant reduction was found in tear secretion by Schirmer tests as compared to BE. Possible explanations as to why no significant reduction in tear secretion was seen after resection of the LG, HG, and NM have been extensively discussed in a separate publication [8].

It has been assumed that accessory LGs are responsible for the remaining tear secretion capacity in the absence of the main LG [6, 15]. However, increasing evidence supports the notion that the conjunctiva can be an important contributor [5, 8, 16, 17]. The accessory LGs are embedded in the conjunctiva, and hence the surface area of conjunctiva is substantially larger than the sum of secreting acinar cell surface area of the accessory LGs. It is not unreasonable to assume that conjunctiva contributes substantial amount to the tear volume in the absence of main LG. Significant difference between SIt and SIIt scores in our rabbit model suggests that sensory regulation of the ocular surface plays an important role. Since accessory LGs have similar functions [18] and innervations as the main LG [19], they are assumed to be under identical reflex control [2]. Although a local transcellular osmotic mechanism is believed to govern the fluid and electrolyte transport [20] fluid secretion by the conjunctiva can also be stimulated [1]. The presence of parasympathetic nerves in rat conjunctiva [21] and increased conjunctiva Cl^−^ and fluid secretion by sympathomimetic agonists [22, 23] suggests that neural influence of conjunctiva secretion cannot be ruled out. And if so, local anesthesia of the secretory nerve terminals could also suppress the secretion output of the conjunctival epithelium. Differences between scores of SIt and SIIt in our study could reflect, at least to a large extent, the basal level tear secretion from accessory LGs and the conjunctiva, whereas it is very difficult, if not impossible, to determine the proportion of contributions from accessory LGs or conjunctiva to the remaining tear secretion capacity.

Contemporary clinical assessments of DE in animal models have certain shortcomings. Tear breakup time and corneal/conjunctiva staining are extremely difficult to evaluate objectively, especially in small animals. Schirmer tests results provide no direct evidence of ocular surface damage. Osmolarity test is expensive and has variable cutoffs [24]. In human, correlations between clinical symptoms, signs of DE, and diagnostic test results have been disappointing as well [25–29]. In our study, poor correlation among the clinical tests (fluorescein staining, rose Bengal staining, and Schirmer tests) is consistent with previous studies. Molecular biomarker based diagnostics, on the other hand, can offer a standardized, objective, and precise measurement of the status of ocular diseases [30] and should be used as adjuncts when possible.

DED associated ocular surface inflammation [31] is caused by increased level of inflammatory cytokines (IL-1, IL-6, TNF-*α*, and IL-17) in tear fluid, corneal/conjunctival epithelia, and increased infiltration of dendritic and T-cells in conjunctiva [32]. In our studies, removal of main LG, HG, and NM led to inflammatory responses at the ocular surface as depicted by increased mRNA [8] and protein levels of TNF-*α* and IL-1*β*. Rabbits with sham surgeries did not show significant increase in biomarker mRNA and protein (data not shown), suggesting that persistent elevation of these markers 1 month AE is not a direct result of surgical procedure itself. Although there was no significant change in tear production at 1 month AE, biomarker evaluations confirmed the increased inflammation which corroborated with the presence of DE phenotype at the ocular surface. Our data is consistent with Solomon et al. who demonstrated that DE is associated with increased production of proinflammatory cytokines (IL-1 and TNF-*α*) in conjunctiva [33]. To the best of our knowledge, overexpression of goblet cell-specific MUC5AC in response to acute DE condition created by surgery is a novel finding in our study. In association with the persistent normal level of tear secretion, MUC5AC overproduction likely contributed to the spontaneous recovery of ocular DE phenotype with time in our rabbit DE model [8]. Gilbard et al. noted reduced conjunctival goblet cell density in their rabbit DE model after cauterizing the LG excretory duct and surgically removing the NM and HG [17], whereas with mucin-specific staining, we were not able to discern any changes in the number or morphology of goblet cells in CIC specimens BE and AE [8]. The exact mechanisms of goblet cell mucin regulations in our rabbit DE model await further investigation.

We isolated both total RNA and protein from CIC specimens, a rapid, convenient, and minimally invasive technique to collect one to three layers of cells from bulbar conjunctival surface [34]. The CIC has been widely performed on subjects to confirm a variety of ocular surface diseases and monitor changes at conjunctival surface. Total RNA and protein isolated from CIC specimen detected subtle changes in mRNA and protein levels of the DED associated cytokines (TNF-*α* and IL-1*β*) and MUC5AC. Biomarkers provided objective and quantitative data that significantly enhanced the characterization of rabbit ocular surface pathology. One CIC specimen per eye at a specific time point offered sufficient high quality total RNA and protein for analyzing several genes without sacrificing the animals. This also enabled us to monitor these rabbits longitudinally and lowered experimental cost [8].

ENaC has been shown to be present in rabbit conjunctiva [35]. Shi and Candia concluded that the electrogenic Na^+^ reabsorption across rabbit conjunctiva was amiloride-insensitive [36], indicating the important roles played by Na^+^ dependent cotransporters such as those carrying glucose and amino acids in series with the basolaterally located Na^+^-K^+^ pump. Hara et al. recently demonstrated increased tear secretion as measured by Schirmer test after the application of amiloride at the rabbit ocular surface [37]. However, we were not able to reproduce their results in our rabbit model. Even using more potent ENaC inhibitor, benzamil [12], no significant increase in tear production was seen in the present study. We concluded that Schirmer test, given its large variation between measurements, may not be sensitive enough to detect subtle change in tear production. Therefore, we further assessed the baseline ocular PD and its response to the application of amiloride in rabbit eyes with and without surgery. Significantly higher (more negative) PD in the operated rabbit eyes was noted in comparison to eyes without surgery. Since electrogenic Cl^−^ secretion and Na^+^ reabsorption across superficial cell apical membrane of the corneal and conjunctival epithelia contribute to the PD [13], the ocular surface tissues must have reached a new equilibrium of higher Cl^−^ secretion and/or Na^+^ reabsorption. Higher magnitude of PD depolarization in the operated eyes in response to the application of amiloride indicates the presence of an elevated amiloride-sensitive Na^+^ conductance (reabsorption) across the epithelia. Although amiloride-insensitive higher Na^+^ reabsorption mechanism could not be measured in the study, it presumably exists. Likewise, a higher Cl^−^ conductance (secretion) most probably is present as well. Our PD measurements demonstrate electrophysiological support of higher tear output across the ocular surface in rabbit eyes without LG, HG, and NM.

To summarize, in this rabbit DED model, although Schirmer tests were unchanged BE and AE, analysis of biomarkers corroborated with the clinical examination findings and confirmed the development of DE condition. Assessing DED pertinent biomarkers enhanced the results obtained from standard clinical tests and is a valuable addition to the tools of ocular surface evaluation. It was interesting to note the elevated MUC5AC expression in the acute DE condition created by surgery but its mechanism requires further investigation. No measurable increased tear secretion was detected with Schirmer test with topical application of amiloride in rabbit eyes AE. However, the open-circuit PD measurement provided a sensitive modality to detect the underlying electrophysiological changes at the rabbit ocular surface AE.
